# Anti-PCSK9 monoclonal antibody attenuates high-fat diet and zymosan-induced vascular inflammation in C57BL/6 mice by modulating TLR2/NF-ƙB signaling pathway

**DOI:** 10.22038/IJBMS.2022.60467.13404

**Published:** 2022-05

**Authors:** Priyanka Arya, Uma Bhandari, Kalicharan Sharma, Priyanka Bansal

**Affiliations:** 1 Department of Pharmacology, School of Pharmaceutical Education and Research (SPER), Jamia Hamdard, New Delhi - 110062, India; 2 Department of Pharmaceutical Chemistry, SPS, DPSRU, New Delhi-110017, India

**Keywords:** Anti-PCSK9 antibody, High-fat diet, Inflammation, Toll-like receptor, Zymosan

## Abstract

**Objective(s):**

Excess intake of a high-fatty diet (HFD) together with zymosan administration mediates vasculitis response which leads to impaired serum lipid levels and causes arterial stiffness. In the development of new cholesterol-lowering medications, PCSK9 inhibitor (proprotein convertase subtilisin/kexin type 9) is an emerging therapeutic. The goal of the present study was to see whether anti-PCSK9 mAb1 might prevent vasculitis in C57BL/6 mice by blocking TLR2/NF-B activation in HFD and Zymosan-induced vasculitis.

**Materials and Methods:**

Protein-protein molecular docking was performed to validate the binding affinity of anti-PCSK9 mAb1 against TLR2. Under the experimental study, mice were randomly allocated to the following groups: Group I: standard mice diet (30 days) + Zymosan vehicle (sterile PBS solution of 5mg/ml on 8^th^ day); Group II: HFD (30 days) + Zymosan ( single IP dose 80 mg/kg on day 8^th^); Group III: HFD+Zymosan + anti-PCSK9 mAb1 (6 mg/kg, s.c. on 10^th^ and 20^th^ days); Group IV: HFD+Zymosan+anti-PCSK9 mAb1 (10 mg/kg, s.c. on 10^th^ and 20^th^ days).

**Results:**

In comparison with the low dose of anti-PCSK9 mAb1 (6 mg/kg), the high dose of anti-PCSK9 mAb1 (10 mg/kg) together with HFD and Zymosan inhibited vasculitis more effectively by decreasing aortic TLR2 and NF-B levels, reducing serum TNF- and IL-6, and up-regulating liver LDLR levels, which down-regulated serum LDL-C and improved serum lipids levels. Histopathological studies showed that anti-PCSK9 mAb1 treatment reduced plaque accumulation in the aorta of mice.

**Conclusion:**

These findings indicate that anti-PCSK9 mAb1 has therapeutic potential in reducing HFD and Zymosan-induced vascular inflammation.

## Introduction

Vascular inflammation is an inflammatory condition of the arteries stimulated by the activation of immune cells or deposition of lipids and proteins to the intimal layer of the arterial walls (1, 2). This, in turn, increases the recruitment of adhesion molecules, cytokines, and chemokine secretion, causing recruitment of immune cells and endothelial cells, which migrate within the vascular endothelium and boost the import of modified lipoprotein particles, finally developing into foam cells (3). The adherence of foam cells results in atherosclerotic plaque formation, which can cause atherosclerosis, resulting, in heart attacks and strokes.

Investigators have reported a substantial relationship between the immune system and metabolic disease-related vascular inflammation (4, 5). Toll-like receptors (TLRs) are innate immune system components that exert a role in the onset and severity of vascular disorders. (6). TLRs are pattern recognition receptors with the primary function of identifying pathogen morphology and controlling infection prevention (7). Earlier studies reported that TLR2 activation leads to activation of specific intracellular signaling factors including nuclear factor-kappa B (NF-ƙB) which triggers an immunological response by releasing inflammatory mediators which lead to endothelium dysfunction, impaired lipid metabolism, unstable plaque, and arterial stiffness (8, 9).

Researchers provide strong evidence that excess consumption of a fatty diet induces an inflammatory response which in turn, alters lipid metabolism (10, 11). Zymosan is a fungal agent that has been long used to induce an inflammatory response that occurs naturally as an insoluble macromolecule, formed from the component of the cell wall of yeast *Saccharomyces cerevisiae* (12, 13). Previous studies have shown that inflammation produced by zymosan in mice promotes the production of proprotein convertase subtilisin/kexin type 9 (PCSK9), which leads to destruction of low-density lipoprotein receptors (LDLR) and an increase in serum LDL-C level which has resulted in lipid dysregulation in C57BL/6 mice (14, 15). Growing data show that zymosan triggers the innate immune system through stimulation of pathogen recognition receptors, and vasculitis is linked to the activation of the TLR2 signaling pathway induced by zymosan. This also conveys the transmembrane signal that causes NF-ƙB to activate (16, 17). NF-ƙB is a major transcription factor that causes production of foam cells and deposition of plaque in the artery wall by inducing inflammatory mediators and altering lipid metabolism by damaging the LDLR. This, in turn, disrupts vascular artery blood flow and raises the risk of atherosclerosis (18). As a result, HFD in addition to zymosan was used as a preclinical model to accelerate vasculitis in mice in this study.

Currently, statins are the most effective therapy for lowering cholesterol and attenuating cardiovascular events (19). However, some patients were not able to tolerate statin therapy due to adverse effects and some indicate the incidence of diabetes mellitus so, novel therapy is required to prevent cardiovascular events (20). PCSK9 inhibitors are an emerging therapy in the development of new cholesterol-lowering drugs (21).

Recently, PCSK9 inhibitors were discovered as a novel therapy for dyslipidemia; preventing LDLR degradation, thus, producing a substantial reduction of LDL-C levels in the bloodstream and decreasing the risk of cardiovascular diseases (22). Investigations have indicated that the impact of anti-PCSK9 mAb1 is not limited to lowering LDL-C but also shows pleiotropic action on immune function, lipoprotein metabolism, platelet aggregation, and thrombosis (23-25). In addition, researchers also reveal that anti-PCSK9 mAb1 treatment reduces the inflammatory response in rheumatoid arthritis and alcohol-induced steatohepatitis (26, 27). However, the effect of anti-PCSK9 mAb1 on vascular inflammation is still unclear. Therefore, the present study hypothesized that anti-PCSK9 mAb1 may reduce the HFD and zymosan-induced dyslipidemia and arterial inflammation.

Anti-PCSK9 mAb1 has not yet been investigated for its role in HFD together with zymosan-induced vasculitis through regulation of the TLR2 and NF-ƙB pathway. Therefore, the current investigation aimed to assess the effects of anti-PCSK9 mAb1 on vascular inflammation in HFD together with zymosan-induced vasculitis via inhibiting the TLR2 and NF-ƙB pathway.

## Materials and Methods

***Drugs and chemicals***

Anti-PCSK9 monoclonal antibody (mAb1) (PL-45134) was obtained as a gift sample by Amgen Inc. Thousand Oaks, California 91320; High-Fat-Diet (HFD consists of fat with about 45% kcal, protein with about 20% kcal, and carbohydrates with about 35% kcal) was offered as a free sample by Ashirwad Industries, Punjab, India (28, 29). Zymosan was bought from Sigma Chemicals.

***In silico study (Protein-protein docking)***

To validate the binding mode of anti-PCSK9 mAb1 at the molecular level, we performed molecular protein-protein docking of anti-PCSK9 mAb1 at the TLR2 level, to perform this we used Maestro, version 10.6 (Schrodinger software). The ligand was drawn in the 3D format using the build panel, and the ligprep tool was utilized for docking. The receptor for the docking study was taken from the TLR2 protein data bank (PDBID: 3a7b) and made by removing the solvent, adding hydrogen, and utilizing the protein preparation wizard to further reduce the energy (30, 31).

Grid for molecular docking was generated by using co-crystallized ligand, LTC **(**(2S)-1-({3-O-[2-(acetylamino)-4-amino-2,4,6-trideoxy-beta-D-galactopyranosyl] alphaDglucopyranosyl}oxy)-3(heptanoyloxy)propan-2-yl(7Z) pentadec-7-enoate) in the receptor (32). The protein-protein docking was validated by withdrawing LTC from the active site of the TLR2 and TLR1 heterodimer and then re-docking it. After confirmation, the anti-PCSK9 mAb1 was docked at the same location. Protein-protein docking (Bioluminate) mode was used to bind anti-PCSK9 mAb1, with up to ten poses stored per molecule. The docking was confirmed by removing LTC from the TLR2/TLR1 heterodimer’s active site and then re-docking it. The anti-PCSK9 mAb1 was docked at the same spot after confirmation. Protein-protein docking (Bioluminate) mode was used to bind anti-PCSK9 mAb1, with each molecule stored in up to ten poses. 

***Animals***

24 Female C57BL/6 mice (9 to 13 weeks of age, weighing 19–20 gm) were taken from Jamia Hamdard, Central Animal House Facility, New Delhi, India after receiving Institutional Animal Ethics Committee (IAEC) approval. The experimental study was done in accordance with Committee for the Purpose of Control and Supervision of Experiments on Animals (CPCSEA) guidelines (Registration no. of JHAEC: 173/GO/Re/S/2000/CPCSEA). Animals were housed in a constant room temperature (22±2 °C) and relative humidity (55±5%) with a 12-hour day/12-hour night cycle, as well as access to food and water *ad libitum****.***

***Study design***

Mice were given HFD pellets randomly for 30 days to induce vasculitis, then zymosan was injected single dosed intraperitoneally (IP) on the eighth day (14). Zymosan (80 mg/kg) solution was prepared by suspending zymosan in sterilized phosphate-buffered saline solution (PBS) to a concentration of 5 mg/ml (13). Based on earlier research, the dose of Zymosan (80 mg/kg, single IP administration) was determined (14, 29). The doses of anti-PCSK9 mAb1 were based on the previous study (22, 33).

Mice were randomly allocated into four groups (n=6 mice per group), after acclimatization: Group I - Normal group: Mice were fed standard mice chow diet fed for a period of 30 days and sterile PBS (zymosan vehicle, 80 mg/kg, *IP*, single injection dose) on the 8^th ^day, Group II - HFD + Zymosan: HFD (30 days) and Zymosan *IP* 80 mg/kg, single dose, day 8, Group III - HFD + Zymosan + anti-PCSK9 mAb1 (6): Mice were administered HFD randomly for 30 days and Zymosan *IP*, 80 mg/kg, a single dose at day 8^th^ + anti-PCSK9 mAb1 were given 6 mg/kg, single s.c. injection x 2 times, i.e., at day 10 and day 20, Group IV- HFD + Zymosan + anti-PCSK9 mAb1 (10) : Mice received HFD randomly for 30 days, Zymosan (80 mg/kg, *IP* single dose at day 8) + anti-PCSK9 mAb1 (10 mg/kg, single s.c. injection x 2 times) i.e., at days 10 and 20. 

On the 31^st^ day, animals were sacrificed, firstly the blood samples were taken from overnight starved mice using retro-orbital plexus puncture for various biochemical analyses. The animals were then euthanized, and aortic and liver tissues were collected, cleaned, and washed in ice-cold normal saline and stored at -80 °C to evaluate the levels of various parameters including aortic TLR2, NF-B, and liver LDLR proteins, respectively. A minute section of the aorta was preserved in a solution of formalin (10%) for histopathological estimation.

***Determination of anthropometric parameters***

Mice food and water intake were measured daily, while their body weight was assessed weekly (34).

***Determination of biochemical parameters***

Span diagnostics kits were used to measure the activities of serum total cholesterol (TC), low-density lipoproteins (LDL), triglycerides (TGs), and high-density lipoproteins (HDL), as per manufacturer’s instructions (35-37). The cardiac risk indices were calculated by the formulas as follows atherogenic index (AI) and coronary risk index (CRI) (LDL-C/HDL-C and TC/HDL-C), respectively (38).

***Determination of aortic TLR2 and NF-*** ƙ***B, and liver LDLR levels***

Using an ELISA kit, the levels of TLR2 and NF-B in ascending aortic tissue and LDLR protein in liver tissue were measured, as per manufacturer’s guidelines (39- 41).

***Determination of*** ^serum^ ***TNF-α and IL-6 levels***

Using an ELISA kit, the levels of TNF-α and IL-6 in serum were measured, as per manufacturer’s guidelines (39, 42).

***Determination of histopathological changes in aorta tissue***

After sacrificing the animal, the aortic tissues were immediately separated from the ascending aorta and stored in a 10% solution of formalin. For histological investigation, the section was stained with hematoxylin (H) and eosin (E) solution. Under a Meiji microscope, the slides were examined (43). The percentage of plaque area in the aorta was determined using Fiji’s Image J software (44). 

***Statistical analysis***

To assess the statistical significance of weekly body weight parameter differences was analyzed of two-way analysis of variance (ANOVA), which was done by Bonferroni’s *post hoc *test. The data were mentioned as mean ± standard error of the mean (SEM). Whereas, one-way ANOVA followed by Tukey’s multiple comparisons test was used to analyze the parameters (Daily food and water intake, inflammatory biomarkers: TLR-2, TNF-α, IL-6, NF-κB, LDLR, cholesterol levels: TC, VLDL, HDL, LDL, TGs, cardiac risk indices, and percentage area of plaque) between the groups, using Graph Pad Prism (5.0) software. The statistical significance level was set at *P*<0.05. 

## Results

***In silico study (molecular protein-protein docking)***

**Figure 1.**
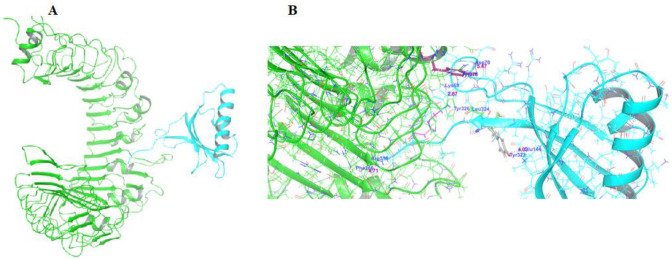
Protein-protein molecular docking of anti-PCSK9 mAb1 against TLR2. (A) Represents the structure of anti-PCSK9 mAb1 (cyano) and TLR2 (Green) ribbon structure. (B) Binding mode of protein-protein interaction of anti-PCSK9 mAb1 (cyano) shown on the TLR2 (Green) protein active site

TLR2 has been shown to have a repeated sequence of leucine-rich amino acids in its active site, with residues including Valine311, Phenylalanin314, Glutamine316, Phenylalanine 312, Glycine313, of TLR1 and Leucine324, Phenylalanine 325, Tyrosine323, Phenylalanine 349, Leucine350, and Tyrosine376 which is mostly involved in TLR2/TLR1 active site (45, 46). The binding of protein, i.e., staphylococcal superantigen like (SSL3) with receptor protein TLR2 is mediated by tyrosine residues in TLR2. Tyr326 promotes SSL3’s inhibitory action towards TLR2 (47). In the current docking investigation, results found that anti-PCSK9 mAb1 showed binding in the active domain including Tyr323, Tyr376, Phe266, and Tyr326 against TLR2 protein (Figure 1).

***Anti-PCSK9 mAb1 effect on intake of feed and water in HFD together with zymosan treated mice***

**Figure 2:**
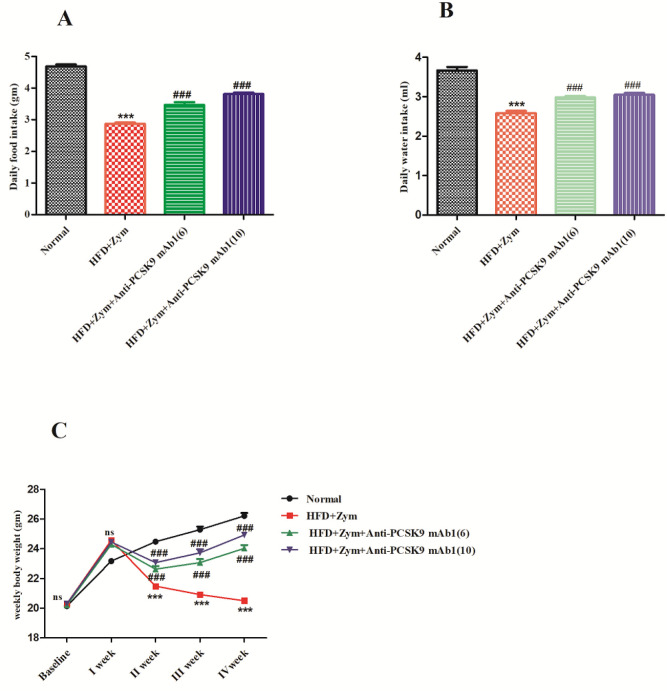
Anti-PCSK9 mAb1 effect on (A) intake of feed, (B) intake of water, and (C) change in body weight per week in HFD and Zymosan-induced vasculitis in mice. ***P<0.001 versus normal group; ###*P*<0.001 and ns *P*>0.05 versus HFD + Zymosan group. Values in the graph are expressed as mean ± SEM (n =6)

In animals treated with HFD together with Zymosan (group II), it was discovered that feeding HFD together with zymosan resulted in a marked (*P*<0.001) reduction in intake of food intake daily in mice in contrast to the standard diet (chow) fed mice. However, when mice were given anti-PCSK9 mAb1 (6 and 10 mg/kg) coupled with HFD together with zymosan for 30 days, there was a substantial (*P*<0.001) rise in intake of food and water found when compared with HFD together with zymosan treated group but less than the normal group (Figure 2A).

Furthermore, when compared with standard diet (chow) fed mice (group I), consumption of HFD combined with zymosan resulted in a substantial (*P*<0.001) decrease in the intake of water in mice (group II). In addition, current research also observed that treatment with anti-PCSK9 mAb1 (6 and 10 mg/kg) resulted in a substantial increase (*P*<0.001) in daily water intake, as compared with HFD together with zymosan groups, but not as much as the normal group. The findings also revealed that a high dose of anti-PCSK9 mAb1 (10 mg/kg) significantly enhanced daily food and water intake compared with a low dose of anti-PCSK9 mAb1 (6 mg/kg) (Figure 2B).

***Effect of anti-PCSK9 mAb1 on body weight per week in HFD together with zymosan treated mice***

Animals fed with the fatty diet for the first week showed a marked (*P*<0.001) rise in body weight (group II). When zymosan (80 mg/kg) was given as a single IP dose on the 8^th^ day and HFD was fed for the next 30 days randomly, there was a substantial (*P*<0.001) reduction in body weight in contrast to the normal group (group I). Anti-PCSK9 mAb1 (6 and 10 mg/kg) treatment in combination with HFD together with zymosan resulted in a significant (*P*<0.001) increase in body weight when compared with HFD and zymosan groups, but less than the normal group (Figure 2C).

***Anti-PCSK9 mAb1 therapy ameliorated serum biochemical levels in HFD together with zymosan treated mice***

Mice on HFD with zymosan (group II) experienced a significant (*P*<0.001) increase in serum TC, LDL-C, VLDL, and TG levels and a significant (*P*<0.001) reduction in serum HDL-C levels when compared with normal mice. Furthermore, when mice were administered with anti-PCSK9 mAb1 (6 and 10 mg/kg) treatment (group III and IV) respectively, along with HFD and Zymosan, serum cholesterol including TC, VLDL, TGs, and LDL-C levels decreased significantly (*P*<0.001) when compared with HFD and zymosan treated mice, i.e., group II. Moreover, serum HDL-C levels increased significantly (*P*<0.001) in anti-PCSK9 mAb1 (6 and 10 mg/kg) treated groups. The results also revealed that a high dose of anti-PCSK9 mAb1 (10 mg/kg) more significantly (*P*<0.001) reduces serum TC, TGs, VLDL, and LDL-C levels and increases HDL-C levels when compared with a low dose of anti-PCSK9 mAb1 (6 mg/kg) (Table 1).

***Effect of anti-PCSK9 mAb1 on the cardiac risk indices in HFD together with zymosan treated mice***

AI and CRI are effective markers for evaluating the risk of atherosclerosis and coronary infarction, as well as detecting the presence of LDL-C in the serum of patients who have been diagnosed with these conditions. The HFD group together with zymosan-treated mice had considerably higher cardiac risk markers (AI and CRI) (*P*<0.001) than the normal group. As the animals were given anti-PCSK9 mAb1 (6 and 10 mg/kg) treatment in addition to HFD and Zymosan, it was discovered that anti-PCSK9 mAb1 medication significantly (*P*<0.001) suppressed HFD and Zymosan-induced rise in AI and CRI when compared with the HFD together with zymosan treated group. Furthermore, it was observed that the high dose of anti-PCSK9 mAb1 (10 mg/kg) treatment together with HFD and Zymosan more significantly (*P*<0.001) reduced cardiac risk indices as compared with the low dose of anti-PCSK9 mAb1 (6 mg/kg) (Table 1).

***Effect of anti-PCSK9 mAb1 on liver LDLR destruction in HFD together with zymosan treated mice***

**Figure 3:**
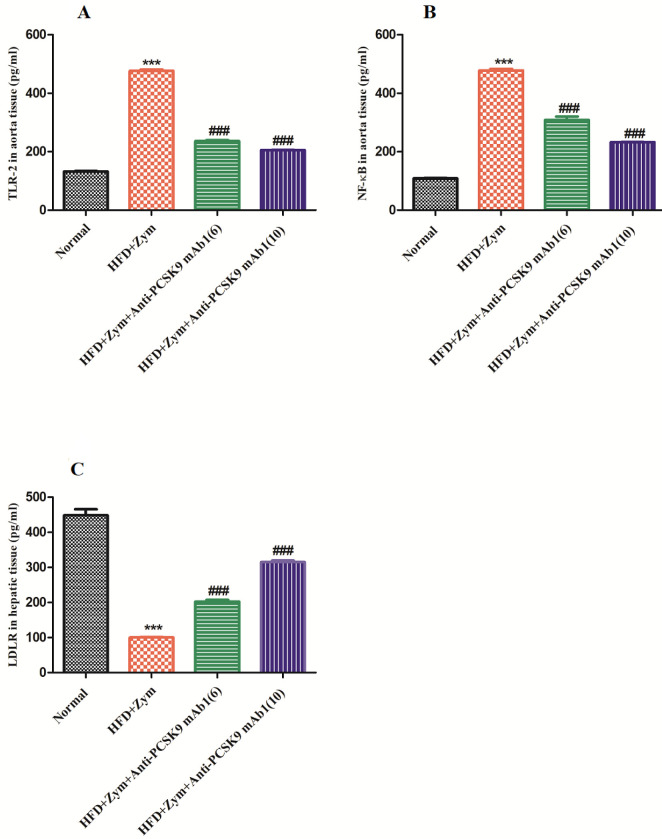
Anti-PCSK9 mAb1 effect on (A) Aortic levels of TLR2, (B) Aortic levels of NF-kB, and (C) Liver levels of LDLR. ***P<0.001 versus normal group; ###*P*<0.001 versus HFD + Zymosan group. Values in the graph are expressed as mean ± SEM (n =6)

Administration of HFD together with zymosan showed a significant (*P*<0.001) reduction in the liver LDLR protein levels in contrast to the normal group. When animals were administered with anti-PCSK9 mAb1 (6 and 10 mg/kg) in addition to HFD together with zymosan, it was observed that this treatment significantly (*P*<0.001) rescued the HFD together with zymosan-caused liver LDLR protein destruction in contrast to HFD together with zymosan treated mice, i.e., group II. As shown in Figure 3C, the high dose of anti-PCSK9 mAb1 (10 mg/kg) treated mice in addition to HFD together with zymosan increased the liver LDLR levels more significantly as compared with the low dose of anti-PCSK9 mAb1 (6 mg/kg) treated C57BL/6 mice (Figure 3C). 

***Effect of anti-PCSK9 mAb1 on aortic TLR2 levels in HFD together with zymosan treated C57BL/6 mice***

The substantial (*P*<0.001) up-regulation in aortic receptor TLR2 levels was observed in the HFD together with zymosan administered mice as compared with the normal group, while a significant (*P*<0.001) decrease in the aortic TLR2 levels was found in the anti-PCSK9 mAb1 (6 and 10 mg/kg) treated groups III and IV, respectively. As the dose of anti-PCSK9 mAb1 increased, the effects on the TLR2 levels tended to become more effective (Figure 3A).

***Effect of anti-PCSK9 mAb1 on aortic NF-*** ƙ***B levels in HFD together with zymosan treated C57BL/6 mice ***

As shown in Figure 3B feeding HFD together with Zymosan group resulted in a significant (*P*<0.001) increase in aortic NF-kB levels in contrast to normal mice, i.e., group I. Anti-PCSK9 mAb1 (6 and 10 mg/kg) treatment together with HFD along with zymosan, showed a significant (*P*<0.001) down-regulation in the aortic NF-kB levels as contrasted to group II. The 10 mg/kg dose of anti-PCSK9 mAb1 treatment led to a reduction in the NF-kB levels more effectively as compared with a low dose of anti-PCSK9 mAb1 (6 mg/kg) treatment.

***Effect of anti-PCSK9 mAb1 on serum pro-inflammatory cytokines in HFD together with zymosan treated C57BL/6 mice***

**Figure 4:**
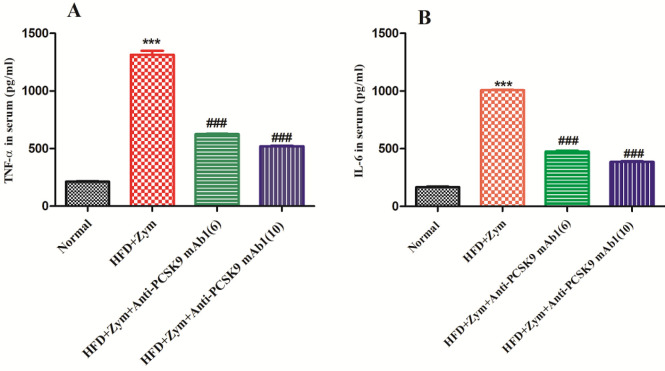
Anti-PCSK9 mAb1 effect on serum pro-inflammatory cytokines (A) TNF-α levels and (B) IL-6 levels in HFD together with Zymosan-induced vasculitis in mice. ***P<0.001 versus normal group; ###*P*<0.001 versus HFD + Zymosan group. Values in the graph are expressed as mean ± SEM (n =6)

Treatment with HFD in addition to zymosan in mice resulted in considerably higher (*P*<0.001) TNF-αand IL-6 levels compared with the normal mice. As anti-PCSK9 mAb1 (6 and 10 mg/kg) was given to mice in groups III and IV, together with HFD in addition to Zymosan, it was discovered that these treatments significantly (*P*<0.001) down-regulated TNF- and IL-6 levels in contrast to the pathogenic group. Furthermore, it was observed that the high dose of anti-PCSK9 mAb1 (10 mg/kg) offered more significant protection (*P*<0.001) against serum TNF-α and IL-6 levels as compared with the low dose of anti-PCSK9 mAb1 (6 mg/kg) (Figures 4A, B).

***Results of histopathological evaluation of the aorta***

**Figure 5:**
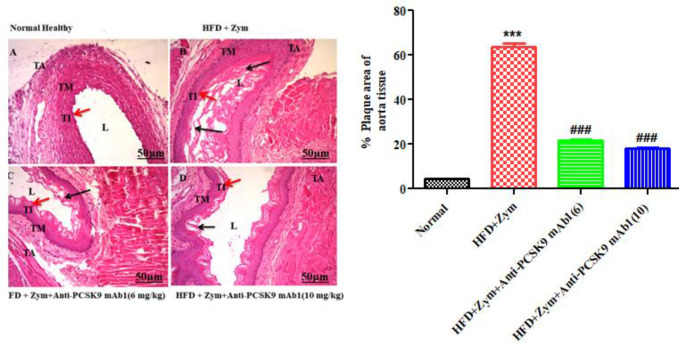
H &E staining photomicrographs of the transverse section of ascending aorta tissues of various groups (10×) [Scale 50 μm]. Note: Normal alignment of the three tunics (L marked for Lumen, TI marked for Tunica intima [Red Arrow], TM marked for Tunica media, TA marked for Lumen Tunica adventitia. Plaque accumulation is presented by the black arrow. (A) Normal (sterile PBS ; Zymosan vehicle), (B) HFD (30 days) and Zymosan (80 mg/kg, single IP dose at day 8^th^), (C) HFD/Zymosan + anti-PCSK9 mAb1 (6 mg/kg, s.c.), (D) HFD/Zymosan + anti-PCSK9 mAb1 (10 mg/kg, s.c.), (E) The percentage area of the atheromatous plaque was calculated (summation of the whole area of atheromatous plaque / whole area of slice) x 100. Image J software was used to determine atherosclerotic plaque area. ***P<0.001 versus normal group; ###*P*<0.001 versus HFD + Zymosan group. Values in the graph are expressed as mean ± SEM (n=6, slides per group)

Figure 5 shows the histopathological changes in various groups. In the normal group, aorta tissue showed a normal structure and no histological changes. As mice were fed an HFD together with zymosan for 30 days, plaque deposition grew substantially (*P*<0.001) in the deepest layer of aortic tissue, the tunica intima layer, resulting in a constriction of the arterial wall of the aorta in contrast to normal mice aorta. When anti-PCSK9 mAb1 (6 and 10 mg/kg) treatments were given together with HFD and zymosan, plaque area in the tunica intima was significantly reduced (*P*<0.001) when compared with the HFD plus zymosan group. In a dose-dependent manner, anti-PCSK9 treated mice produced a significant reduction in plaque formation in the aorta.

## Discussion

Vascular inflammation causes a variety of serious arterial problems, including atherosclerosis, circulatory disease, hemorrhage, and peripheral arterial disease, which account for the majority of cardiovascular morbidity and mortality. Therefore, inhibition of the arterial inflammatory process is a promising target for preventing and treating arterial events and complications (18).

Current research tries to evaluate the role of anti-PCSK9 mAb1 in mitigating the HFD and Zymosan-induced vasculitis and its possible underneath mechanism. Administration of HFD together with zymosan to mice for a period of 30 days, resulted in a marked increase in the TLR2, transcription factor NF-ƙB, and cytokines TNF-α and IL-6 levels and a decrease in the LDLR level which was eventually reversed by anti-PCSK9 mAb1 therapy.

It was observed that animals in the HFD and zymosan group on high-calorie intake with the usage of HFD alone without zymosan for a week showed a marked increase in their anthropometric parameters, compared with the mice that were fed a normal diet. 

On the other hand, administration of a single dose of zymosan (80 mg/kg, intraperitoneal injection) on the 8^th^ day and continuous feeding of HFD diet for 23 days (all together with a 30 days treatment of HFD) induced vasculitis in mice, which exerted a remarkable decrease in body weight per week, feed and water intake compared with the standard diet (chow) fed mice. Our results are similar to the findings of Zhang *et al*. who reported that rats were inactive and showed a drop in food and water consumption (48). Similar trends were confirmed in the study of Malik *et al*. who reported that when zymosan was given to C57BL/6 mice it caused inflammatory reactions, resulting in catabolic conditions in mice in the first 24 hr (13).

In addition, this study found that treatment with anti-PCSK9 mAb1 (6 and 10 mg/kg) together with HFD and Zymosan resulted in an increase in weekly bodyweight and daily food and water intake when compared with the mice treated with HFD and Zymosan but less than the standard diet (chow) fed group. Hence, the present investigation confirmed that treatment with anti-PCSK9 mAb1 improves the food consumption up to the normal diet.

The TLR2 and NF-kB pathway is one of the imperative pathways in the pathophysiology of vasculitis because it involves the activation of a cascade of inflammatory mediators (49). TLR is a transmembrane protein in the immune system that can identify a variety of pathogenic related molecules and serves as an effective barrier for the body’s immune system to resist pathogens (50). TLR2 is activated by pathogenic molecules, which activate the intracellular signaling protein NF-κB resulting in generation of cytokines such as TNF-α, IL-1β, and IL-6. These changes led to endothelium dysfunctioning and cause the production of foam cells and plaque instability (49, 51). Further, this led to the development of metabolic diseases like dyslipidemia and atherosclerosis. Edfeldt *et al*. observed that activation of TLR2 and NF-κB led to development of atherosclerotic plaques in humans (52). Madam and colleagues reported that administration of HFD along with Porphyromonas gingivalis in mice triggers an arterial inflammatory response by increasing aortic TLR2 levels (4).

The current study evaluated whether the anti-inflammatory effect of anti-PCSK9 mAb1 (6 and 10 mg/kg) in arterial inflammation is regulated by the TLR2 and NF-kB cascades or not. As a result, we examined TLR2 and NF-kB levels in the aorta of mice. HFD and zymosan administration elevated vasculitis by increasing aortic TLR2 and NF-kB levels, which resulted in a rise in the percentage of plaque area in the aorta, as validated by histological examination of the aorta. In addition to HFD and zymosan, anti-PCSK9 mAb1 (6 and 10 mg/kg) therapy lowered TLR2 and NF-kB levels, as well as plaque development in the aorta of mice. Our findings are consistent with those of Bhaskar and colleagues wherein they reported that quercetin treatment inhibits TLR2 and NF-kB levels which resulted in reducing the atherosclerotic inflammation (53). The present study demonstrated that anti-PCSK9 mAb1 can attenuate vascular inflammation through suppression of TLR2/NF-κB expression. The inhibitory effect of anti-PCSK9 mAb1 on the TLR2/NF-κB signaling pathway was demonstrated for the first time, which is the novel finding of our study.

Using molecular docking, we confirm the hypothesized protein-protein binding of anti-PCSK9 mAb1 to the TLR2 receptor protein and further investigate whether anti-PCSK9 mAb1 alters the pattern recognition receptors. *In silico* studies have found that anti-PCSK9 mAb1 links with TLR2 protein through catalytic binding residues Tyr323, Tyr376, Phe266, and Tyr326 against TLR2. As a result, the current investigation discovered that anti-PCSK9 mAb1 has a binding affinity for TLR2 protein. Therefore, the present study identified anti-PCSK9 mAb1 as a potential modulator of TLR2 protein. 

In addition, previous studies have shown that TLR2 triggers the secretions of inflammatory agents (such as TNF-α and IL-6) through activation of NF-kB signaling and develops the atherosclerotic process (4). Furthermore, we discovered in the present research work that mice fed HFD and Zymosan had abnormally high levels of TNF- and IL-6, whereas animals given anti-PCSK9 mAb1 (6 and 10 mg/kg) had significantly lowered levels. However, a high dose of anti-PCSK9 mAb1 (10 mg/kg) has a greater effect on reducing the levels of inflammatory mediators. Our findings are consistent with prior research wherein the researchers had reported a reduction in arterial inflammation by decreasing the level of pro-inflammatory agents (54).

Inflammation mediates multiple alterations in lipid and lipoprotein metabolism (14). LDLR is a protein that is abundantly expressed in the liver and is responsible for maintaining lipid metabolism. LDLR degradation is closely associated with up-regulation of TLR2 and NFƙB levels, causing the release of TNFα and IL6 and leading to hepatic LDLR degradation (55). Degradation of this tightly regulated LDLR pathway allows LDL-C to accumulate intracellularly, resulting in foam cell production. Foam cells that accumulate in vascular endothelium provoke a vasculitis, which in turn accelerates lipid buildup and promotes arterial inflammatory reactions (56, 57).

Previous studies have reported that HFD, along with Zymosan in C57BL/6 mice, induced inflammation, stimulated PCSK9 expression, increased hepatic LDLR degradation, and thereby increased serum LDLC cholesterol levels (14). Interestingly, our results showed a dramatic drop in LDLR levels in the liver, increased serum LDLC levels, and also showed changes in the lipid metabolism chart (marked rise in serum TC, VLDL, and TG levels and drop in serum HDL-C levels) in mice treated with HFD in addition to zymosan for a period of 30 days. Furthermore, using anti-PCSK9 mAb1 together with HFD and zymosan dramatically boosted liver LDLR levels, resulting in lower LDL cholesterol levels. Furthermore, as compared with HFD together with zymosan-only administered mice, anti-PCSK9 mAb1 treatment reduced TC, VLDL, and TG levels, as well as cardiac risk indices, and dramatically improved HDL-C levels. The present study provides strong evidence that the anti-PCSK9 mAb1 treatment, in addition, to rescuing the LDLR degradation, also modulates cholesterol and TG content.

The results of the histological study confirmed the aforementioned findings, revealing that the group treated with HFD together with Zymosan had increased atheromatous plaque area percentage in the intima of the aortic tissue. Treatment with anti-PCSK9 mAb1 (6 and10 mg/kg) resulted in a reduction in atheromatous plaque area percentage, while a high dose of anti-PCSK9 mAb1 exerted greater efficacy as compared with a low dose of anti-PCSK9 mAb1.

**Table 1:** Effect of anti-PCSK9 mAb1 in serum biochemical levels in HFD and Zymosan induced vasculitis in mice

**Groups**	**TC ** **(mg/dl)**	**TGs (mg/dl)**	**HDL (mg/dl)**	**LDL (mg/dl)**	**VLDL (mg/dl)**	**AI** **= (LDL-C/HDL-C)**	**CRI** **= (TC/HDL-C)**
Normal	113 ± 1.18	65.8 ± 0.92	46.3± 0.15	53.5 ± 0.96	13.1 ± 0.18	1.15 ± 0.19	2.44 ± 0.02
HFD+Zymosan	252.8 ± 0.38^a^	261.5 ± 0.53^a^	15.7 ± 0.30^a^	184.7 ± 0.49^a^	52.3 ± 0.10^a^	11.7 ± 0.23^a^	16.05 ± 0.29^a^
HFD/Zymosan+Anti-PCSK9 mAb1(6)	196 ± 0.11^b^	126 ± 0.73^ b^	36.7 ±0.15^ b^	134 ± 0.30^ b^	25.28 ± 0.14^ b^	3.64 ± 0.02^ b^	5.331 ± 0.02^ b^
HFD/Zymosan+Anti-PCSK9 mAb1(10)	156 ± 1.00^ b,c^	94.5 ± 0.93^ b,c^	39.7 ±0.11^ b,c^	97.7 ± 0.94^ b,c^	18.90 ± 0.18^ b,c^	2.45 ± 0.02^ b,c^	3.935 ± 0.03^ b,c^

Values are expressed as mean ± SEM (n = 6 animals per group). a*P*<0.001 versus normal group; b*P*<0.001 versus HFD+Zymosan group; c*P*<0.001 vs HFD/Zymosan+Anti-PCSK9 mAb1(6) group

## Conclusion

The present investigation provides strong evidence that arterial inflammation was caused by feeding HFD together with zymosan administration for 30 days in C57BL/6 mice. Anti-PCSK9 mAb1 (6 mg/kg and 10 mg/kg, s.c.) treatment can effectively prevent arterial inflammatory reactions caused by HFD together with zymosan in mice by improving the lipids levels and also reducing cardiac risk factors/indices. Furthermore, anti-PCSK9 mAb1 treatment significantly inhibited the levels of pro-inflammatory agents TLR2, NF-ƙB, TNF-α, and IL-6, and increased LDLR levels. However, high dose anti-PCSK9 mAb1 (10 mg/kg, s.c.) treatment offered more protective effect than low dose of anti-PCSK9 mAb1, i.e., 6 mg/kg, s.c., against vascular inflammation. In conclusion, our research showed for the first time that the anti-PCSK9 mAb1 (6 and 10 mg/kg) treatment attenuated the vasculitis via modulating the TLR2 and NF-ƙB pathway in HFD together with zymosan treated mice.

## Authors’ Contributions

PA and UB Study conception and design; PA Perform experiment; KC *In silico* analysis; PA, PB, UB Analysis and interpretation of results; PA and UB Manuscript writing; PA and UB Critical revision or editing of the article; PA, PB, UB Final approval of the version to be published; UB Guided and supervised the research.

## Conflicts of Interest

None.
